# ESG rating disagreement and bank loan availability: Evidence from China

**DOI:** 10.1371/journal.pone.0317191

**Published:** 2025-01-06

**Authors:** Jidong Qin, Meijia Wang

**Affiliations:** Business School, Chengdu University of Technology, Chengdu, China; University of Kotli Azad Jammu and Kashmir, PAKISTAN

## Abstract

Environmental, social, and governance (ESG) ratings are receiving increasing attention in credit markets. However, ESG rating disagreement erects obstacles for companies in obtaining capital resources. This study investigates the impact of ESG rating disagreement on bank loan availability uses data of Chinese listed firms from 2014 to 2022, and employs models with multiple regression analyses and fixed effects. We find that greater ESG rating disagreement leads to a decrease in newly obtained bank loans. The mechanism analysis confirms that ESG rating disagreement amplifies information asymmetry and increases operational uncertainty, thereby raising the information and credit risks faced by banks, leading to a decrease in bank loan availability. Heterogeneity analysis reveals that the negative effect of ESG rating disagreement on bank loan availability is more pronounced in firms with poor financing capabilities, poor information environments, and fierce competitive macro environments. Our findings contribute to the literature on ESG rating disagreement from credit markets, which are important for a more comprehensive and objective understanding of ESG rating disagreement.

## 1. Introduction

In recent years, sustainable development incorporating environmental, social, and governance (ESG) factors has attracted significant attention worldwide. According to the Global Sustainable Investment Alliance (GSIA), total global sustainable investment reached approximately $30.3 trillion in 2022. In China, the “dual carbon goal” of achieving the carbon peak by 2030 and carbon neutrality by 2060 aims to attain a sustainable economy, and listed companies play an important role in achieving this goal. By mid-2024, approximately 37% of Chinese A-share listed companies had disclosed their ESG reports for 2023. Simultaneously, numerous market participants make decisions based on companies’ ESG performance [1]. Therefore, ESG factors have become a priority for capital markets, enterprises, and governments.

Both practitioners and scholars have attempted to measure and compare the ESG performance of companies using the ESG ratings provided by third-party rating agencies. In China, approximately 20 rating agencies provide ESG rating information to market participants. However, owing to the lack of unified ESG disclosure and evaluation standards, significant differences in rating methodologies, frameworks, and weights among various rating agencies result in rating disagreements for a single company [2–4]. Berg et al. [1] found that for a company, the correlation coefficient among credit ratings published by various agencies reached 0.99, whereas the correlation coefficient among ESG ratings ranged only from 0.38 to 0.71. For instance, in 2022, SynTao Green Finance rated the ESG performance of Kweichow Moutai as “C+”, while Sino-Securities rated it “AA”, illustrating a stark disagreement of ESG rating.

The ESG rating disagreement introduces uncertainty and inconsistency in the financial market, incurring additional costs for capital market participants. Such divergence in ESG ratings creates confusion among investors and may potentially mislead information users, thus reducing resource allocation efficiency in capital markets [5]. Therefore, exploring the economic consequences of ESG rating disagreements is essential. Currently, research on the economic consequences of ESG rating disagreement is limited. Existing studies have primarily explored the impact of ESG rating disagreement on portfolio management, asset pricing, and stock returns [6, 7], focusing primarily on the stock market, the influence of ESG rating disagreement on the credit market remains underexplored. Banks are a crucial capital source for companies in emerging markets [8], and ESG performance is becoming increasingly important for companies to obtain loans from banks [9]. China’s ESG development is in the early stages, and the economic consequences of ESG rating disagreement are unclear [10]. Therefore, examining the impact of ESG rating disagreement on bank loan availability in China is crucial for efficient capital resource allocation and companies’ sustainable development.

Therefore, this study explores the relationship between ESG rating disagreement and bank loan availability in China, contributing to a deeper understanding of the role of ESG information in capital resource allocation in emerging markets. We selected Chinese A-share listed companies from 2014 to 2022 as the research sample and used data from five rating agencies: Wind, SynTao Green Finance, Sino-Securities, FTSE Russell, and SusallWave. The empirical results show that ESG rating disagreement significantly affects bank loan availability, the greater ESG rating disagreement, the smaller the newly obtained bank loans. The mechanism analysis confirms that ESG rating disagreement amplifies information asymmetry and increases operational uncertainty, thereby increasing the information and credit risks faced by banks, ultimately leading to a decrease in loan size. We further analyzed heterogeneity in terms of the following three aspects: firm characteristics, information environment, and macro environment. The results reveal that ESG rating disagreement has a greater negative impact on firms with poor financing capabilities, poor information environments, and fierce competitive macro environments.

This study contributes to existing literature in several ways. First, it expands the research on the impact of ESG information on bank loans. Existing studies have investigated the impact of ESG performance on bank loans. However, they have used the overall ESG scores or detailed dimensions (environment, social, or governance) from a single rating agency. Additionally, they have not accounted for ESG rating disagreement among various agencies. Our study enhances the understanding of ESG information based on the economic consequences of rating disagreement among different agencies. Second, it enriches the research on the economic consequences of ESG rating disagreement from a credit market perspective. Prior research has primarily focused on the economic consequences of the stock market, such as portfolio management, asset pricing, and stock returns. By examining the impact of ESG rating disagreement on bank loans, we offer novel insights into the economic consequences of ESG rating disagreement in the credit market. Third, this study provides empirical evidence about the impact of ESG disagreement on emerging capital markets. Most studies have focused on developed Western capital markets, whereas our study focuses on the impact of ESG rating disagreement on bank loan decisions in China. Therefore, our study not only helps to deepen the understanding of ESG rating disagreement but also offers insights from emerging capital markets.

## 2. Literature review and hypothesis development

### 2.1 ESG rating disagreement

The research on ESG rating disagreement has focused on two areas: contributions and economic consequences. The ESG rating disagreement is fundamentally attributed to rating agencies and enterprises. From the rating agency perspective, there is a lack of consensus on rating methodologies, measurements, and weights of evaluation criteria among rating agencies [1, 2]. Additionally, individual raters’ subjective interpretations, influenced by their varying information collection and processing abilities, exacerbate companies’ ESG rating divergence [5]. From the enterprise perspective, the non-mandatory nature of ESG reports results in differences in disclosure policies and content among companies. Voluntary ESG disclosure that complies with the Global Reporting Initiative (GRI) and obtains third-party certification helps mitigate rating disagreement [11]. Conversely, ESG reports with a more positive tone or ambiguous expression can amplify ESG disagreements.

The ESG rating disagreement has several economic consequences. First, the divergence reduces ESG information effectiveness, Billio et al. [4] and Serafeim and Yoon [12] found that ESG rating disagreement reduces the predictive power of ESG information on stock prices, resulting in lower return on investments based on ESG principles. Second, the hidden uncertainty leads investors to demand heightened risk compensation [3], which significantly impacts stock returns [6, 7]. Additionally, high uncertainty affects capital resource allocation, and greater ESG disagreement is related to higher financing costs [5]. Finally, ESG rating disagreement, as a proxy for information asymmetry, negatively impacts innovation activities [13] and analysts’ forecast quality [14].

### 2.2 Bank loan availability

Theoretically, the firms’ availability of bank loan is predominantly influenced by information and credit risk [15]. Regarding information risk, banks use enterprises’ financial and non-financial information, the quantity and quality of which significantly impact firms’ bank loan availability [16]. Previous studies have extensively examined the effects of financial information on bank loan size, revealing that high-quality financial reports can reduce information asymmetry between companies and banks, enabling favorable bank loan decisions [17–19]. Non-financial information, such as CSR reports, ESG reports, and R&D disclosures, can also minimize information asymmetry between companies and banks [20]. Previous studies have examined the relationship between borrowers’ ESG information and bank loans from various perspectives, such as pricing, maturity, covenants, and loan size [21–23]. Additionally, some studies have examined the effect of detailed ESG dimensions on bank loans [24, 25].

Regarding credit risk, companies’ operational performance uncertainty affects future free cash flows, which directly relates to firms’ repayment ability [26, 27]. Within an enterprise, equity structure [28], internal controls [29], tax avoidance activities [30], and innovation activities [31] are directly related to operational performance uncertainty and credit risk, further influencing bank loan availability. Additionally, according to stakeholder theory, enterprise–stakeholder relationships directly influence operational performance. Companies that have stronger relationships with stakeholders, such as the government [32], banks [33], suppliers, and customers [34], tend to exhibit superior operational performance and obtain more favorable loan covenants from banks.

### 2.3 Hypothesis development

Previous studies have identified information and credit risks as crucial mechanisms affecting the bank loan availability [15]. We elucidate how ESG rating disagreement affects companies’ ability to obtain bank loans from two perspectives: information and credit risk.

First, ESG rating disagreement exacerbates the information asymmetry between banks and enterprises, thereby increasing information risk. Information asymmetry occurs when one party in a transaction has more or better information than another. According to signaling theory, enterprises’ voluntary disclosure can offer incremental information [35]. The ESG reports disclose the measures implemented by companies in the environmental, societal, and corporate governance dimensions, reflecting their sustainable development capability. Thus, the ESG information increases banks’ understanding of enterprises’ true situation, reduces information asymmetry [36], and helps enterprises obtain better bank loan contracts [23, 37]. However, in contrast to ESG performance, ESG rating disagreement reduces the reliability of ESG rating information, creating ambiguity about a firm’s true ESG performance for external capital providers [13]. In the context of bank loan decisions, ESG rating disagreement increases banks’ costs and exacerbates information asymmetry between banks and firms. Therefore, we argue that ESG rating disagreement enhances the information risk associated with information asymmetry and decreases banks’ willingness to lend.

Second, ESG rating disagreement increases operational performance uncertainty, reduces free cash flow, and increases credit risk. According to stakeholder theory, stakeholders are crucial for enterprises, as they can help companies obtain lower financing costs, increase stable material supply, and enhance revenue sources, thereby reducing operational uncertainty and enhancing operational performance [38]. Enterprises with better ESG scores are considered to be less risky and tend to receive help from stakeholders. For example, Apergis et al. [37] found that firms with higher ESG scores have lower bond spreads and better bond ratings. Bagh et al. [39] found that ESG performance has an inverse U-shaped relationship with firms’ sustainable growth. Conversely, The ESG disagreement makes it more challenging for stakeholders to discern enterprises’ true situation, leading to increased risk assessment. Additionally, investors reduce their investments and demand higher risk compensation from companies with greater ESG rating disagreement [3, 6]. Thus, ESG rating disagreement reduces stakeholder support, increases uncertainty in operational performance and free cash flow, and makes loan recovery challenging for banks. This, in turn, heightens the credit risk faced by banks. Therefore, we argue that ESG rating disagreement increases operational performance uncertainty and credit risk, and reduces banks’ willingness to lend. Based on the above analysis, we propose the following hypothesis:

***Hypothesis 1*: *Ceteris paribus*, *ESG rating disagreement will decrease bank loan availability*.**

## 3. Sample selection and research design

### 3.1 Sample selection

This study examines the impact of ESG rating disagreement on loan availability using Chinese A-share listed companies from 2014 to 2022. Our data come from the annual ESG ratings of five agencies: Wind, SynTao Green Finance, Sino-Securities, FTSE Russell, and SusallWave. For each ESG ratings agency, the Sino-Securities ESG rating data starts from 2011, the SusallWave ESG rating data starts from 2014, the SynTao Green Finance ESG rating data starts from 2015, the FTSE Russell and Wind ESG rating data both start from 2018. To ensure that each listed company has rating data from at least two rating agencies, our research period starts from 2014. Following convention, we excluded samples from the financial sector, those with less than one year of listing, samples under special conditions (ST and *ST), as well as samples with missing data. To eliminate the influence of extreme values, all continuous variables were winsorized at the 1% to 99% level. Ultimately, 16,699 firm-year observations were obtained. The ESG rating data were derived from the Wind database, while company data were obtained from the CSMAR database.

### 3.2 Variable definition and research design

#### 3.2.1 ESG rating disagreement

The dependent variable in our study is ESG rating disagreement. Following Avramov et al. [3], we use the standard deviation of ESG ratings from different agencies to measure ESG rating disagreement. The specific steps are as follows: (1) Initial data processing. Unify the ESG ratings of listed companies by five rating agencies into a standardized format to ensure comparability among rating agencies. (2) Sort processing. Annually rank the ESG scores of enterprises evaluated by each rating agency. The higher the score, the higher the ranking. Enterprises with the same score receive the same ranking. (3) Standardized processing. Standardize the enterprise rankings of various rating agencies using the range normalization method, and ensure that all ESG ratings range between 0 and 1. (4) Form paired rating disagreement. Calculate the standard deviation of the standardized ranking of a company by every two rating agencies as paired rating disagreement. Finally, take the average of all paired rating differences as the ESG rating disagreement (*ESGD*).

#### 3.2.2 Bank loan availability

The dependent variable in our study is bank loan availability. Following Wang et al. [40], we used the scale of newly obtained bank loans in the current period to represent bank loan availability and standardized the newly obtained bank loans by the initial total assets. (*dLoan*).

#### 3.2.3 Model setting

To investigate the impact of the ESG rating disagreement on bank loan availability, we referred to previous studies [31, 40] and set the following model for empirical testing:

$$\begin{array}{c} {dLoan}_{i,t}=\beta_{0}+\beta_{1}{ESGD}_{i,t}+\beta_{2}{Size}_{i,t}+{\beta_{3}{Roe}_{i,t}+\beta }_{4}{Lev}_{i,t}+\beta_{5}{Cash}_{i,t}+\beta_{6}{Tang}_{i,t}\\ +\beta_{7}{Tq}_{i,t}+\beta_{8}{Age}_{i,t}+\beta_{9}{Top1}_{i,t}+\beta_{10}{Indir}_{i,t}+\beta_{11}{Dual}_{i,t}+\beta_{12}{Soe}_{i,t}\\ +\beta_{13}{Big4}_{i,t}+{Year}_{t}+{Industry}_{j}+\varepsilon_{i,t} \end{array}$$ (1)

Where *dLoan* is the dependent variable: bank loan availability, which is calculated as the newly obtained bank loans scaled by initial total assets. *ESGD* is the independent variable, measured by the average of the paired standard deviations of ESG ratings by different rating agencies. Following Liang et al. [31] and Wang et al. [40], we also account for various factors that might influence a corporation’s debt financing ability. These primarily include: firm size (*Size*), return on equity (*ROE*), debt to asset ratio (*Lev*), cash flow (*Cash*), proportion of tangible assets (*Tang*), firm growth (*T*q), listing years (*Age*), ownership concentration (*Top*1), proportion of independent directors (*Indir*), duality (*Dual*), property rights (*SOE*), and audit quality (*Big*4). In addition, we control for the year-fixed effect (*μ*_*t*_) and industry-fixed effect (*γ*_*j*_). The detailed variable definitions are shown in Table 1.

**Table 1:** Definition of variables.

Variables	Symbol	Definition
Dependent Variable	*dLoan*	Newly obtained loans are divided by the initial total assets of the period.
Independent Variable	*ESGD*	ESG rating disagreement, detailed calculation process can be found in 3.2.1
Controls	*Size*	Natural logarithm of total assets
	*Roe*	Net profit divided by net assets
	*Lev*	Total liabilities divided by total assets
	*Cash*	Operating cash flow divided by total assets
	*Tang*	Tangible assets divided by total assets
	*Tq*	Tobin Q value
	*Age*	Natural logarithm of listing years
	*Top1*	The shareholding ratio of the largest shareholder
	*Indir*	The proportion of independent directors to all directors
	*Dual*	1 = CEO duality; 0 = CEO non-duality
	*Soe*	1 = state-owned enterprises; 0 = non state-owned enterprises
	*Big4*	1 = Big4 audit firms; 0 = non Big4 audit firms

## 4. Empirical results and analysis

### 4.1 Descriptive statistics and correlation analysis

#### 4.1.1 Descriptive statistics

The descriptive statistics of the main variables are shown in Table 2. We can find that the minimum value of the dependent variable *dLoan* is 0.0006, the maximum value is 0.3214, the mean is 0.0217, and the standard deviation is 0.0748. This indicates a significant difference in the availability of bank loans for different enterprises, and most enterprises find it difficult to obtain bank loans. The minimum value of the independent variable *ESGD* is 0.0038, the maximum value is 0.5250, the mean is 0.1840, and the standard deviation is 0.1180, indicating substantial differences in ESG rating disagreement among enterprises. The statistical results of the other variables are generally consistent with existing literature, showing no significant anomalies.

**Table 2:** Descriptive statistics.

Variable	N	Mean	SD	Min	p50	Max
*dLoan*	16699	0.0217	0.0748	0.0006	0.0081	0.3214
*ESGD*	16699	0.1840	0.1180	0.0038	0.1590	0.5255
*Size*	16699	22.6003	1.3759	20.1289	22.3907	26.6665
*Roe*	16699	0.0492	0.1596	-0.7988	0.0693	0.3764
*Lev*	16699	0.4540	0.1890	0.0907	0.4483	0.8984
*Cash*	16699	0.0496	0.0655	-0.1419	0.0479	0.2447
*Tang*	16699	0.2040	0.1530	0.0021	0.1720	0.6694
*Tq*	16699	2.3180	1.5730	0.8350	1.8327	9.6380
*Age*	16699	3.0310	0.2810	2.1972	3.0445	3.5835
*Top1*	16699	0.3311	0.1481	0.0826	0.3049	0.7366
*Indir*	16699	0.3790	0.0540	0.3333	0.3636	0.5714
*Dual*	16699	0.3020	0.4593	0	0	1
*Soe*	16699	0.3300	0.4701	0	0	1
*Big4*	16699	0.0777	0.2677	0	0	1

#### 4.1.2 Correlation analysis

The Pearson correlation matrix of the main variables is reported in Table 3. The results show that the Pearson correlation coefficient of *ESGD* and *dLoan* is −0.038 and significant at the 1% level, which indicates that firms with higher ESG rating disagreement acquire fewer newly obtained bank loans. This provides preliminary evidence that ESG rating disagreement is negative to bank loan availability, which is consistent with our basic hypothesis. In addition, as observed from the results, the range of the correlation coefficients between explanatory variables is reasonable from −0.304 to 0.401, excluding the possibility of multicollinearity between variables and demonstrating the validity of the parameter estimation.

**Table 3:** Correlation matrix.

	*dLoan*	*ESGD*	*Size*	*Roe*	*Lev*	*Cash*	*Tang*	*Tq*	*Age*	*Top1*	*Indir*	*Dual*	*Soe*	*Big4*
*dLoan*	1													
*ESGD*	-0.038***	1												
*Size*	0.026***	-0.005	1											
*Roe*	0.092***	-0.071***	0.151***	1										
*Lev*	0.136***	0.081***	0.459***	-0.247***	1									
*Cash*	-0.251***	-0.040***	0.099***	0.338***	-0.161***	1								
*Tang*	-0.014*	-0.022***	0.105***	0.0120	0.018**	0.251***	1							
*Tq*	0.006	-0.032***	-0.348***	0.123***	-0.307***	0.126***	-0.142***	1						
*Age*	-0.083***	0.021***	0.111***	-0.047***	0.126***	-0.009	0.033***	-0.143***	1					
*Top1*	0.008	-0.008	0.230***	0.157***	0.046***	0.106***	0.117***	-0.095***	-0.064***	1				
*Indir*	-0.008	0.001	0.002	-0.012	0.006	-0.003	-0.038***	0.050***	-0.030***	0.039***	1			
*Dual*	0.060***	-0.017**	-0.199***	-0.003	-0.112***	-0.027***	-0.085***	0.148***	-0.121***	-0.071***	0.113***	1		
*Soe*	-0.083***	0.023***	0.401***	0.01	0.241***	0.000	0.150***	-0.240***	0.209***	0.272***	-0.048***	-0.304***	1	
*Big4*	-0.009	-0.064***	0.364***	0.069***	0.096***	0.072***	0.038***	-0.045***	-0.020***	0.159***	0.040***	-0.055***	0.138***	1

This table reports the Pearson correlations between main variables. ***, **, * indicate significance at the 1%, 5% and 10% levels, respectively.

### 4.2 Benchmark regressions

We employed Model (1) to examine the impact of ESG rating disagreement on bank loan availability, the regression results are shown in Table 4. Column (1) presents the regression results without the control variables, while column (2) includes the control variables. The regression coefficients of *ESGD* in columns (1) and (2) are -0.0222 and -0.0224, respectively, and are both significant at the 1% level. This represents that with every one-unit increase in the standard deviation of ESG rating disagreement, the average growth rate of newly obtained bank loans decreases by 12.07% (0.0222 × 0.1180 / 0.0217) and 19.18% (0.0224 × 0.1180 / 0.0217) of the sample mean. This suggests that greater ESG rating disagreement leads to decrease in bank loan availability, which aligns with our basic hypothesis.

**Table 4:** Benchmark regressions.

	(1)	(2)
	*dLoan*	*dLoan*
*ESGD*	-0.0222***	-0.0224***
	(-4.3795)	(-4.7352)
*Size*		0.0008
		(1.2113)
*Roe*		0.1113***
		(23.7021)
*Lev*		0.0909***
		(19.5575)
*Cash*		-0.3873***
		(-32.3988)
*Tang*		0.0168***
		(3.3023)
*Tq*		0.0029***
		(6.3111)
*Age*		-0.0167***
		(-6.9081)
*Top1*		0.0001**
		(2.1044)
*Indir*		-0.0197*
		(-1.8807)
*Dual*		0.0052***
		(3.8582)
*Soe*		-0.0174***
		(-12.0629)
*Big4*		-0.0016
		(-0.6848)
*_cons*	0.0258***	0.0291*
	(22.5781)	(1.7736)
*Year*	Yes	Yes
*Industry*	Yes	Yes
*N*	16699	16699
*adj*. *R*^*2*^	0.1150	0.1682

Note: *, **, *** are significant at 10%, 5%, and 1%, respectively; The value of t is in parentheses, the following tables are the same.

Concerning control variables, the coefficients for *Roe*, *Tang*, and *Tq* are statistically positive, suggesting that firms with higher profitability, more mortgageable assets, and higher growth opportunities can obtain more bank loans [40, 41]. The coefficients for *Cash*, *Age*, and *Soe* are statistically negative, indicating that firms with more cash flow, larger listing years, and owned by the state are less likely to obtain capital resources from banks [31]. Overall, the coefficients on control variables in Table 4 are largely consistent with the prior literature.

### 4.3 Robustness tests

#### 4.3.1 Endogenous test: Instrumental variable

Bank loan availability contains information that can serve as a reference for rating agencies, thereby affecting companies’ ESG rating. Thus, our findings may be affected by the reverse causality problem. First, we used an exogenous policy shock to address endogeneity. In 2019, the Hong Kong Stock Exchange (HKSE) reformed ESG disclosure requirements, issuing a revised version of the “ESG Reporting Guide” which expanded the scope of mandatory disclosure. This reform helps reduce irregularities in ESG information disclosure, thereby decreasing ESG rating disagreements among rating agencies. We constructed a dummy variable, *HK*, which equals 1 if the company is also listed and traded on the HKSE, and 0 otherwise. Additionally, we constructed another dummy variable, *Post*, which equals 1 if the year is 2019 or later, and 0 otherwise. We used the interaction term *HK*×*Pos*t of these two dummy variables as an instrumental variable, positing that enterprises listed on both the Chinese A-share market and HKSE are more affected by the policy change, resulting in lesser ESG rating disagreements. The regression results of this instrumental variable are presented in columns (1) and (2) of Table 5. The regression coefficient of *HK*×*Post* is significantly negative, indicating that the HKSE’s policy changes have indeed reduced ESG rating disagreements. In column (2), the regression coefficient of *ESGT_hat* is significantly negative, indicating that larger ESG rating disagreement corresponds to lower bank loan availability, which is consistent with the benchmark regression results.

**Table 5:** Instrumental variable method.

	(1)	(2)	(3)	(4)
	*ESGD*	*dLoan*	*ESGD*	*dLoan*
HK*Post	-0.0348***			
	(-2.7239)			
*HK*	-0.0451***			
	(-3.5544)			
*ESGDM*			0.9733***	
			(14.2315)	
*ESGD_hat*		-0.0234***		-0.0240***
		(-4.9178)		(-5.0084)
*Controls*	Yes	Yes	Yes	Yes
*_cons*	0.2368***	0.0226	0.1096***	0.0226
	(7.4561)	(1.3832)	(3.2083)	(1.3823)
*Year*	Yes	Yes	Yes	Yes
*Industry*	Yes	Yes	Yes	Yes
*N*	16699	16699	16699	16699
*adj*. *R*^*2*^	0.1473	0.1683	0.1532	0.1683

Second, considering the industry spillover effect of ESG information disclosure [42], we selected the mean ESG rating disagreements within the same industry and year (*ESGDM*) as another instrumental variable. The regression results of this instrumental variable are presented in columns (3) and (4) of Table 5. In column (3), the regression coefficient of *ESGDM* is significantly positive, indicating a positive correlation between the company’s ESG rating disagreement and the industry ESG rating disagreement. In column (4), the regression coefficient of *ESGD_hat* is significantly negative, indicating that larger ESG rating disagreements correspond to lower bank loan availability, which is consistent with the benchmark regression results. Overall, the regression results in Table 5 indicate that, after mitigating the potential reverse causality problem, the main conclusions of this study still hold.

#### 4.3.2 Endogenous test: PSM method

We employed the Propensity Score Matching (PSM) to address potential sample selection bias. Specifically, we used the median ESG rating disagreement (*ESGD*) as the benchmark. Samples with *ESGD* values above the median are assigned a value of 1, and 0 otherwise. Using all control variables in Model (1) as matching variables, we applied nearest-neighbor matching (1:1) to select matched samples and re-tested the basic hypothesis. Table 6 presents the regression results after PSM sample selection. The regression coefficients of *ESGD* in columns (1) and (2) remain negative and significant at the 1% level. This indicates that, even after addressing potential endogeneity problems caused by sample selection bias, the main conclusions of this study still hold.

**Table 6:** PSM method.

	(1)	(2)
	*dLoan*	*dLoan*
*ESGD*	-0.0199***	-0.0183***
	(-2.9459)	(-2.9566)
*Controls*	No	Yes
*_cons*	0.0254***	0.0096
	(16.1860)	(0.4342)
*Year*	Yes	Yes
*Industry*	Yes	Yes
*N*	8874	8874
*adj*. *R*^*2*^	0.1129	0.1718

#### 4.3.3 Other robust tests

*(1) Replace the measurement of the dependent variable*. In the benchmark regression, we use the newly obtained bank loans divided by the initial assets as the dependent variable. To ensure the robustness of the empirical results, we alternatively use the ratio of newly obtained bank loans to operating revenue to re-measure the bank loans (*dLoan1*). The regression results are shown in Table 7, column (1). The regression coefficient of *ESGD* remains significantly negative, indicating that our main finding remains valid even after changing the measurement of the dependent variable.

**Table 7:** Other robust tests.

	(1)	(2)	(3)	(4)	(5)	(6)
	*dLoan1*	*dLoan*	*dLoan*	*dLoan*	*dLoan*	*dLoan*
*ESGD*	-0.0356***			-0.0136**	-0.0177***	-0.0225***
	(-2.9328)			(-2.3394)	(-3.1033)	(-4.7379)
*ESGDif*		-0.0040***				
		(-3.7819)				
*ESGR*			-0.0026***			
			(-4.5913)			
*L*.*dLoan*					0.0708***	
					(6.4443)	
*GDP*						0.0006**
						(2.0557)
*dCredit*						0.0012
						(1.5450)
*Controls*	Yes	Yes	Yes	Yes	Yes	Yes
*_cons*	0.0210	0.0230	0.0075	-0.3659***	-0.0111	0.0259
	(0.5329)	(1.4070)	(0.4399)	(-3.3104)	(-0.6115)	(1.5276)
*Year*	Yes	Yes	Yes	Yes	Yes	Yes
*Industry*	Yes	Yes	Yes	Yes	Yes	Yes
*Firm*	No	No	No	Yes	No	No
*N*	16699	16699	16699	16156	12260	16699
*adj*. *R*^*2*^	0.1123	0.1678	0.1681	0.2794	0.1787	0.1683

*(2) Replacing the measurement of independent variable*. In the benchmark regression, we used the mean value of the standard deviation of ESG ratings from paired agencies. Here, we use the standard deviation of ESG ratings from all agencies to measure ESG rating disagreement (*ESGDif*) and the range of ESG ratings from all agencies (*ESGR*). The regression results are shown in Table 7, columns (2) and (3). The regression coefficients of *ESGDif* and *ESGR* are both significantly negative, indicating that our main finding remains valid after changing the measurement of the independent variable.

*(3) Replacing the regression model*. In the benchmark regression, we controlled only for year-fixed effects and industry-fixed effects. To control for more potential variables that do not vary with enterprise characteristics, we further controlled for firm-fixed effects. The regression results are shown in Table 7, column (4). The regression coefficient of *ESGD* remains significantly negative, indicating that our main finding remains valid after replacing the regression model.

*(4) Controlling the previous bank loans*. Banks refer to previous loan decisions when making current loan decisions. Therefore, we introduce the previous bank loans (*L*.*dLoan*) as a control variable in Model (1). The regression results are shown in Table 7, column (5). The regression coefficient of *ESGD* remains significantly negative, indicating that our main finding remains valid after controlling for previous bank loans.

*(5) Controlling the macroeconomic variables*. Bank loan decisions are influenced not only by corporate characteristics but also by macroeconomic factors [43]. Therefore, we introduce the GDP growth rate(*GDP*) and social credit growth rate(*dCredit*) to control the impact of macroeconomic factors. The regression results are shown in Table 7, column (6). The regression coefficient of *ESGD* remains significantly negative, indicating that our main finding remains valid after controlling for macroeconomic factors.

### 4.4 Further analysis

#### 4.4.1 Mechanism analysi

In the theoretical analysis, we delineated two channels that elucidate the ramifications of ESG rating disagreement on bank loan availability. Primarily, ESG rating disagreement accentuates information asymmetry between enterprises and banks, thereby augmenting the information risk. Additionally, ESG rating disagreement signals heightened companies’ operational performance uncertainty, thereby elevating the credit risk. To verify the soundness of the aforementioned theoretical analysis, we constructed the following model.


$${Med}_{it}=\beta_{0}+\beta_{1}{ESGD}_{i,t}+\beta_{n}{Controls}_{i,t}+\mu_{t}+\gamma_{j}+\varepsilon_{i,t}$$ (2)

Where *Med* represents the mediation variable, and *Controls* denote the control variables, which remain consistent with Model (1).

To investigate the information risk mechanism, we examined the impact of ESG disagreement on information and disclosure quality. Specifically, information quality was measured as the absolute value of discretionary accruals(*DA*), estimated using the modified Jones model [44, 45], in which a higher *DA* indicates lower information quality. The disclosure quality was assessed using the disclosure assessment results (*Disc*) published by the Shenzhen Stock Exchange and Shanghai Stock Exchange, where a higher *Disc* value reflects better disclosure quality [46]. The results of the information risk mechanism are presented in columns (1) and (2) of Table 8. In column (1), the regression coefficient of *ESGD* is significantly positive, indicating that greater ESG rating disagreement leads to lower information quality. In column (2), the regression coefficient of *ESGD* is significantly negative, suggesting that higher ESG rating disagreement results in lower disclosure quality. This validates the information risk mechanism proposed in the theoretical analysis, indicating that larger ESG rating disagreements exacerbate information asymmetry between enterprises and banks.

**Table 8:** Mechanism analysis.

	(1)	(2)	(3)	(4)
	*DA*	*Disc*	*ZScore*	*Sdroa*
*ESGD*	0.0135**	-0.1891***	-0.1049**	0.0080**
	(2.1854)	(-4.4440)	(-1.9798)	(2.3394)
*Controls*	Yes	Yes	Yes	Yes
*_cons*	0.0210	0.0230	0.0075	-0.3659***
	(0.5329)	(1.4070)	(0.4399)	(-3.3104)
*Year*	Yes	Yes	Yes	Yes
*Industry*	Yes	Yes	Yes	Yes
*N*	16699	16699	16699	16699
*adj*. *R*^*2*^	0.1547	0.1545	0.1125	0.1156

To investigate the credit risk mechanism, we examined the impact of ESG disagreement on financial and operational risks. Specifically, financial risk was determined using Altman’s Z-Score Model, in which a higher *Z-Score* signifies lower financial risk [47]. Operational risk was measured as the standard deviation of profits over the past three years (*Sdroa*), with a higher *Sdroa* indicating greater operational risk [48]. The results of the credit risk mechanism are displayed in columns (3) and (4) of Table 8. In column (3), the regression coefficient of *ESGD* is significantly negative, suggesting that greater ESG rating disagreement leads to higher financial risk. In column (4), the regression coefficient of *ESGD* is significantly positive, indicating that higher ESG rating disagreement corresponds to higher operational risk. This validates the credit risk mechanism proposed in the theoretical analysis and highlights that larger ESG rating disagreements increase uncertainty regarding a company’s future operation performance and decrease the likelihood of banks recovering loans.

#### 4.4.2 Heterogeneity tests

*(1) Heterogeneous effects of firm characteristics*. The ESG rating provided by agencies and bank loan availability can vary depending on the firm’s characteristics, and the negative effect of ESG rating disagreement on bank loan availability may vary with firms’ characteristics. Therefore, we consider the heterogeneous effects of the following three firm characteristics.

**①Profitability.** When making loan decisions, banks focus on whether firms can repay a loan on schedule. Firms with higher profitability have stronger future solvency [49]. Therefore, when profitability is low, the negative effect of ESG rating disagreement on bank loan availability is expected to be stronger. To verify this, we divided the samples into two groups based on median profitability (*Roe*) and regressed Model (1) separately. The specific regression results are shown in columns (1) and (2) of Table 9. The coefficient of *ESGD* in the high profitability group is not significant, whereas the coefficient of *ESGD* in the low profitability group is significantly negative, and the difference between the two groups is significant at the 10% level. This indicates that when a firm’s profitability is low, the negative effect of ESG rating disagreement on bank loan availability is stronger.

**Table 9:** Heterogeneity of firm characteristics.

	(1)	(2)	(3)	(4)	(5)	(6)
	*High profitability*	*Low profitability*	*SOEs*	*non-SOEs*	*Heavily-polluting enterprises*	*Non-heavily-polluting enterprises*
*ESGD*	-0.0051	-0.0186***	-0.0034	-0.0336***	-0.0336***	-0.0115*
	(-0.6332)	(-3.1231)	(-0.4394)	(-5.6655)	(-3.5432)	(-1.8756)
*Controls*	Yes	Yes	Yes	Yes	Yes	Yes
*_cons*	0.0532**	0.0522**	0.0062	0.0052	0.0441	0.0509**
	(2.0500)	(2.2815)	(0.2293)	(0.2410)	(1.1292)	(2.4263)
*P-value*	0.0557*		0.0017***		0.0488**	
*Year*	Yes	Yes	Yes	Yes	Yes	Yes
*Industry*	Yes	Yes	Yes	Yes	Yes	Yes
*N*	8348	8351	5507	11192	4323	12376
*adj*. *R*^*2*^	0.1374	0.1625	0.1530	0.1802	0.1849	0.1610

**②Property Rights.** Firms’ property rights are a decisive factor when obtaining bank loans. Compared to state-owned enterprises (SOEs), non-state-owned enterprises (non-SOEs) find it more challenging to obtain bank loans [50]. Therefore, the negative effect of ESG rating disagreement on bank loan availability is expected to be stronger for non-SOEs. To verify this, we divided the samples into two groups based on property rights and regressed Model (1) separately. The specific regression results are shown in columns (3) and (4) of Table 9. The coefficient of *ESGD* in the SOEs group is not significant, whereas the coefficient of *ESGD* in the non-SOEs group is significantly negative. The difference between the two groups is significant at the 5% level, indicating that for non-SOEs, the negative effect of ESG rating disagreement on bank loan availability is stronger.

**③Industry.** ESG principles focus on sustainability issues such as environmental and social responsibility. Therefore, compared to other industries, ESG ratings are more important for heavily-polluting industries, and the negative effect of ESG rating disagreement on bank loan availability is expected to be stronger in these industries. To verify this, we divided the samples into two groups based on whether firms belong to heavily-polluting industries [51] and regressed Model (1) separately. The specific regression results are shown in columns (5) and (6) of Table 9. For heavily-polluting enterprises, the coefficient of t *ESGD* is -0.0336 and significant at the 1% level, and for non-heavily-polluting enterprises, the coefficient of *ESGD* is -0.0115 and significant at the 10% level. The difference between the two groups is significant at the 5% level. This indicates that for heavily-polluting enterprises, the negative effect of ESG rating disagreement on bank loan availability is stronger.

*(2) Heterogeneous effects of information environment*. The information environment can alter the negative effects of ESG rating disagreement on bank loan availability. The theoretical analysis indicated that ESG rating disagreement exacerbates information asymmetry, and increases the information risk faced by banks, thereby leading to a reduction in bank loan availability. Therefore, when the information environment is transparent and comprehensive, banks have a better understanding of the company’s true situation. Conversely, a poor information environment leads to a stronger negative effect of ESG rating disagreement on bank loan availability. To validate this analysis, we used three intermediary criteria to measure the information environment: audit quality (*Big4*), analyst coverage (*Analyst*), and media coverage (*Media*). When audit quality is high, analyst and media coverage is more, and the information environment is better [52–54]. We then divided the samples into two groups based on the median quality of the information environment and regressed Model (1) separately. The specific regression results are shown in Table 10.

**Table 10:** Heterogeneity of the information environment.

	(1)	(2)	(3)	(4)	(5)	(6)
	*Big4*	*Non-Big4*	*More analyst coverage*	*Less analyst coverage*	*More media coverage*	*Less media coverage*
*ESGD*	-0.0226	-0.02857***	-0.0071	-0.0208***	-0.0033	-0.0217***
	(-1.4440)	(-4.6225)	(-0.8758)	(-3.3629)	(-0.3906)	(-3.6831)
*Controls*	Yes	Yes	Yes	Yes	Yes	Yes
*_cons*	0.0172	0.0270	0.0516*	0.1029***	0.0348	0.0466**
	(0.3579)	(1.5186)	(1.6944)	(4.0954)	(1.2019)	(2.0023)
*P-value*	0.0794*		0.0715*		0.0632*	
*Year*	Yes	Yes	Yes	Yes	Yes	Yes
*Industry*	Yes	Yes	Yes	Yes	Yes	Yes
*N*	1298	15401	8357	8342	8392	8307
*adj*. *R*^*2*^	0.1358	0.1713	0.1640	0.1441	0.1647	0.1684

Table 10 shows that when audit quality is high, analyst and media coverage is more, the coefficient of *ESGD* is not significant. Whereas, when audit quality is low, analyst and media coverage is lower, the coefficient of *ESGD* is significantly negative. This indicates that when the quality of intermediary information is lower, in which the company’s information environment is poor, the negative effect of ESG rating disagreement on bank loan availability is stronger.

*(3) Heterogeneous effects of macro environment*. The macro environment can also alter the negative effects of ESG rating disagreement on bank loan availability. When market competition is high, bank loans to support firms are more imperative [55]. Therefore, the negative effect of ESG rating disagreement on bank loan availability is expected to be stronger in a more competitive market. To verify this analysis, we calculated the Herfindahl-Hirschman Index (HHI) of the markets, the smaller the HHI, the greater the market competition. Then we divided the samples into two groups based on the annual median HHI and regressed Model (1) separately. The specific regression results are shown in columns (1) and (2) of Table 11. The results indicate that when market competition is low, the coefficient of *ESGD* is not significant, whereas when market competition is high, the coefficient of *ESGD* is significantly negative. This indicates that in a more competitive market, the negative effect of ESG rating disagreement on bank loan availability is stronger.

**Table 11:** Heterogeneity of the macro-environment.

	(1)	(2)	(3)	(4)	(5)	(6)
	*High HHI*	*Low HHI*	*High FMGI*	*Low FMGI*	*High bank density*	*Low bank density*
*ESGD*	-0.0077	-0.0246***	-0.0086	-0.0218***	0.0014	-0.0185***
	(-1.0271)	(-3.5786)	(-1.0944)	(-3.3495)	(0.1487)	(-2.7486)
*Controls*	Yes	Yes	Yes	Yes	Yes	Yes
*_cons*	0.0614**	0.0344	0.0052	0.0657***	0.5881***	0.0310
	(2.0119)	(1.5954)	(0.1713)	(2.9210)	(2.7586)	(1.3545)
*P-value*	0.0920*		0.0956*		0.0589*	
*Year*	Yes	Yes	Yes	Yes	Yes	Yes
*Industry*	Yes	Yes	Yes	Yes	Yes	Yes
*N*	8278	8421	8418	8281	8355	8344
*adj*. *R*^*2*^	0.1559	0.1874	0.1626	0.1570	0.1401	0.1576

Additionally, Competition in the financial environment is a crucial factor influencing bank loans. When competition in the financial environment is low, banks dominate loan negotiations and impose stricter requirements on companies. Conversely, banks become more lenient with increasing competition, firms can obtain loans more easily. This implies that the negative effect of ESG rating disagreement on bank loan availability is expected to be more pronounced in a less competitive financial environment. To verify this analysis, we used the financial market growth index(FMGI) and local bank density to measure the degree of competition in the financial environment [56, 57], divided the samples into two groups based on the degree of competition, and regressed Model (1) separately. The specific regression results are shown in Table 11, columns (3) to (6). The results show that when the financial market growth index and local bank density are high, the coefficient of *ESGD* is not significant, and when the financial market growth index and local bank density are low, the coefficient of *ESGD* is significantly negative. This indicates that in a less competitive financial environment, the negative effect of ESG rating disagreement on bank loan availability is stronger.

## 5. Conclusion

With the increasing focus on green and sustainable development, firms’ ESG performance has garnered significant investor attention. Nonetheless, the lack of unified ESG disclosure standards and varying rating agency capabilities have led to ESG rating disagreements. Despite this, research on how ESG rating disagreement affects stakeholder support for enterprises is limited. Therefore, we investigated the impact of ESG rating disagreement on bank loan availability from a perspective of credit market, using ESG rating data from Chinese A-share listed companies from 2014 to 2022.

Our results show that ESG rating disagreement significantly decreases firms’ newly obtained bank loans, indicating that companies with higher ESG rating disagreement have a lower bank loan availability. After controlling for endogenous problems and a series of robustness tests, the results remain robust. The mechanism test verifies that ESG rating disagreement exacerbates information asymmetry and increases operational uncertainty, thereby increasing information and credit risk faced by banks, and ultimately leading to a decrease in bank loan availability. Heterogeneity analysis revealed that the impact of ESG rating disagreements on bank loan availability varies with firm characteristics, information environment, and macro environment. Specifically, the negative effect is stronger for firms with lower profitability, non-SOEs, and in highly-polluting industries. This negative effect is amplified when intermediary information quality, such as audit quality, analyst coverage, and media coverage is poor. Additionally, this negative effect is more pronounced when market competition is high, the regional financial development index is low, and local bank density is low.

Our findings have important implications for three key participants in the capital market. First, the government should strive to establish standardized and regulated ESG disclosure standards, which enhance the information content and comparability of ESG ratings, thereby reducing the adverse effects of ESG rating disagreement. Second, listed companies should actively implement green development initiatives and fulfill their social responsibilities, thereby increasing transparent voluntary disclosures related to ESG practices and enhancing the positive impact of ESG information. Finally, banks are encouraged to enhance their ability to collect and process ESG information and focus on firms’ ESG practices rather than agencies’ ESG ratings. By doing so, they can mitigate the disruption caused by ESG rating disagreement on their loan decisions, thereby improving capital resource allocation efficiency.

This study has some limitations. We only considered the impact of overall ESG disagreement, our analysis did not consider the impact of specific ESG dimensions. Future research could investigate the economic consequences of the specific ESG dimensions’ rating disagreement. In addition to the amount of bank loans, there are various loan contractual terms such as loan maturity, structure, and collateral. Whether and how ESG rating disagreement affects other bank loan features is a topic for future exploration.
