# Correction: ESG rating disagreement and bank loan availability: Evidence from China

**DOI:** 10.1371/journal.pone.0351570

**Published:** 2026-06-10

**Authors:** Jidong Qin, Meijia Wang

The Funding statement for this article is incorrect. The correct Funding statement is as follows: This study was funded by the Philosophy and Social Science Research Fund of Chengdu University of Technology (Grant No. YJ2024-QN010). The funders had no role in study design, data collection and analysis, decision to publish, or preparation of the manuscript.
